# Increase in Search Interest for “Suicide” and “Depression” for Particular Days of the Week and Times of Day: Analysis Based on Google Trends

**DOI:** 10.3390/jcm12010191

**Published:** 2022-12-26

**Authors:** Jacek Stańdo, Żywilla Fechner, Agnieszka Gmitrowicz, Karl Andriessen, Karolina Krysinska, Adam Czabański

**Affiliations:** 1 Centre of Mathematics and Physics, Lodz University of Technology, 90-924 Lodz, Poland; 2 Institute of Mathematics, Lodz University of Technology, 93-590 Lodz, Poland; 3 Department of Child and Adolescent Psychiatry, Medical University of Lodz, 90-674 Lodz, Poland; 4 Melbourne School of Population and Global Health, The University of Melbourne, Melbourne, VIC 3010, Australia; 5 Department of Social Sciences, Jacob of Paradies University, 66-400 Gorzow Wielkopolski, Poland

**Keywords:** suicide, suicide attempt, depression, Google Trends

## Abstract

Depression is the most common disorder in people who attempt suicide or die by suicide. Research review indicate that therapy of depression (including psychoeducation) is one of the main factors in the prevention of suicidal behavior. In this paper we examine the intensification of search interest for the terms “depression” and “suicide” in Google search engine with regard to the time of day and day of the week in Poland, Germany, Great Britain and Italy. The goal of the study was to determine if there are any days of the week or hours when search for “suicide” and “depression” particularly increases. Numerous studies focusing not only on the seasonality of suicidal behavior, but also on the days of the week and hours, indicate that it is most often undertaken on Mondays in the night and early morning hours. The results of the research being the basis of this paper show a certain time coincidence: first, the increase in search interest for “suicide” and “depression” and then undertaking suicidal behavior (suicide and suicide attempts). Searching for terms “suicide” and “depression” usually took place (except in Italy) at weekends and most often in the late evening hours and at night. The conclusions from the research can be used in suicide prevention activities, for example in determining the hours of operation of individual helpline numbers.

## 1. Introduction

According to WHO data (2014, 2019, 2021 https://www.who.int/publications/i/item/9789241564779, https://www.who.int/publications/i/item/9789240026643, https://www.who.int/publications/i/item/9789240026629, Accessed date: 21 November 2022), despite many activities in the field of suicidal behavior prevention in various areas of public health and science, the number of suicides worldwide exceeds 700,000 annually. Monitoring data on the number of suicides and suicide attempts is the basis for assessing the effectiveness of preventive measures. Among the various groups of factors determining suicidal behavior, the relationship between their occurrence and time is of profound significance, including the season of the year, time of day, public holidays, and the day of the week.

Research on the days of the week and the time of suicide attempts in adolescents in Poland, showed that the highest number of suicide attempts occurred on Mondays (27%), followed by Thursdays (21%), Sundays (17%), Wednesdays (15%) Fridays (9%), Tuesdays (8%) and the lowest on Saturdays (3%). The highest proportion of suicide attempts occurred in the evening hours (35%) [1], pp. 76–83. Research conducted on a nationwide sample confirmed earlier findings from Poland (M. Makara-Studzińska; K. Rosa [2]); A. Polewka et al. [3], pp. 269–273; K. Bliźniewska-Kowalska et al. [4], pp. 44–49; B. Hołyst [5], pp. 377–378 and [6], pp. 34, 226–235, and showed that in the years 1999-2011 the highest number was recorded between 2 p.m. and 9:59 p.m. and the lowest at night (from 10 p.m. to 5:59 a.m.). Moreover, it was found that suicidal behaviors were mostly undertaken on Mondays (B. Hołyst [5], pp. 36, 247–256). Suicide attempts were most common in the fall months (October, November), and the least frequent in February and May. The research findings regarding the seasonality of suicide in Poland shows that the number of suicides declines in the fall and winter months, and increases in the spring and summer months, starting in March and ending in August. This trend is particularly strong in July (130% compared to the monthly average) and in January (76.9% compared to the monthly average) the average (A. Młodożeniec et al. [7], pp. 61–69). The analysis based on police data (website: National Police Headquarters) also reveals that in the years 2019–2021 in Poland, the highest number of suicides was recorded in May, June, and July (from 109.6% to 119.9% of the average annual rate). The lowest frequency of suicides occurred in February (85.2% to 88.1% of the average annual suicide rate).

Studies in other countries, provide further data on the frequency of suicide and suicide attempts in particular months of the year, days of the week and hours of the day. Studies in Scandinavia and Greece show an increase in the number of suicides in spring and early summer (T. Partonen et al. [8], pp. 133–139; E. Petridou et al. [9], pp. 106–109). Studies in Australia, America and South Korea show that the number of suicides increases in spring and decreases in winter (B.M. Roehner [10], p. 350–362; P.G. Dixon et al. [11], pp. 66, 685–697; C.T. Yang et al. [12]). A study in northern Bavaria showed that the number of suicide attempts increased in spring and decreased in December. Further, the risk of women attempting suicide in spring was 13.1% higher than in other seasons and this seasonality of suicide attempts was not statistically confirmed in the male population (R. Mergl et al. [13], pp. 393–400). Moreover, other German studies show that suicide by a specific method—on railroad tracks—is most often recorded in April and September (N. Erazo et al. [14], pp. 1–9; N. Erazo et al. [15], pp. 137–143; K. Lukaschek et al. [16], p. 124). Recent research on the seasonality of suicides in Germany in 2019-2020 demonstrates in 2019 the most suicides were recorded in April and July, while in 2020 the most suicides were recorded in May, June and July (T. Schelhase [17], pp. 3–10).

Earlier multicentre studies conducted in the years 1990–1992 centers indicated that the number of both suicides and suicide attempts increased in spring, while their occurrence declined in December and the following winter months. However, this relationship was found only among women (G. Jessen et al. [18], pp. 57–69). Australian studies covering 81 different latitudes have confirmed that there are links between gender and the seasonality of suicides. Studies have found an increase in the number of suicides in men in late spring and early summer, while in women an increases are observed in spring and autumn (C. Law et al. [19], pp. 2825–2833).

Many studies suggest that suicide rates are higher during periods of high temperatures (Helama et al. [20]; Dixon et al. [21]). Preti [22]) claimed that climate variables could explain 63% of suicides in Italy. In Kazakhstan, an increase in the average temperature by 1 degree Celsius was associated with an increase in the number of suicides by 2.1 (Grjibovski [23]). Similarly, in a study in Finland, temperature variability explained more than 60% of the total suicide variance (Helama et al. [20]). In turn, in Taiwan (Tsai [24]), a negative correlation of suicides with temperature was observed. These studies found no link between rising temperatures and suicide rates. Studying the seasonality of suicides in relation to temperature variability was not the purpose of this study.

Research also indicates fluctuation in the incidence if suicide and attempted suicide around holidays. A study in Hungary found connections between the dynamics of suicides and the holiday season over 1970–2022. According to the authors, during the holidays people may have more time to reflect on their lives, make a balance sheet of their life, which may contribute to a decision to die. A large number of suicides happen on New Year’s Day, while on Christmas Day and on Easter Saturday and Monday, the number of suicides among men is lower, which is referred to as “Gabennesch’s broken promise”, that is, the effect of the day after the holiday. Despite this observation, another study in Hungary found no impact of national holidays on the frequency of suicide (T. Zonda [25], pp. 153–162). On the other hand, in Australia, researcher found that the number of suicides increased in a week before and a week after public holidays (J.C. Lawes et al. [26]). Research in Japan over 1979-1994 showed that the rate of suicides was the lowest on the days before a holiday and the highest on the days after a holiday (M. Nishi et al. [27], pp. 317–320).

Studies, which explored the relationship between suicide attempts and suicides and a specific day of the week, identified some similarities and differences between countries. In Japan, the greatest number of suicides were recorded on Mondays while Saturdays had the lowest incidence (M. Nishi et al. [27], pp. 317–320). In Australia, regardless of the season, men were most likely to die by suicide on Mondays; this result was not found for women (C. Law et al [19], pp. 2825–2833). Other studies also found that the peak days for suicide rates were Mondays (18.3%) and Saturdays (16.4%) (J.C. Lawes et al. [26]). In Austria, the highest number of suicides was found on Mondays and Tuesdays (E.A. Deisenhammer et al. [28], pp. 93–100), while the lowest rate was observed at weekends (M. Plöderl [29], pp. 438–445). In Italy, most suicides were recorded on Mondays (A. Pretti et al. [30], pp. 253–261). In Germany, most suicides on the railroad happen on Mondays and Tuesdays (N.Erazo et al. [14], pp. 1–9; N. Erazo et al. [15], pp. 137–143; K. Lukaschek et al. [16], p. 124). A multicentre study in 13 European countries showed that suicides were most often observed on Mondays, while suicide attempts were most common on Sundays and Fridays; this phenomenon applied mostly to women (G. Jessen et al. [18], pp. 57–69).

Studies also looked at hourly fluctuations in the incidence of suicide and suicide attempts. Overall, findings reveal that suicide attempts are more frequent late in the evening, while suicides took place during the day. The knowledge that suicide attempts tend to occur mostly in the evening or early at night can inform development of preventive actions, especially telephone crisis helplines (G. Jessen et al. [18], pp. 57–69). In Italy, daily fluctuations in occurrence of suicides were found in both men and women, and an increase in the number of suicides was observed in the early morning and at night. Further, among adolescents, the peak in the number of suicides was in late afternoon, while adults aged 25–44 died by suicide mostly from the morning until the early afternoon hours. This finding may be related to the daily rhythm of work and school activities of family members, who may not be present at home at certain times (A. Pretti [30], pp. 253–261). In Austria, suicides occur most frequently from the morning until noon (M. Plöderl [29], pp. 438–445). In Germany, suicides on railroad tracks are most frequent between 6.00 a.m. and noon and between 6.00 p.m. and midnight (N. Erazo et al. [14], pp. 1–9; N. Erazo et al. [15], pp. 137–143; K. Lukaschek et al. [16], p. 124). The late evening and night hours may be chosen due to the reduced vigilance of relatives. In addition, on weekdays (especially on Mondays), suicides happen at such times when there is nobody at home.

The highest risk of suicidal behavior is noted in patients with recurrent depression and depression in bipolar affective disorder. Various statistics speak of a 15–20 percent risk of suicide in people suffering from depression. Suicide is more than 20 times more common in these conditions than in the general population. (Romaszko [31], pp. 315–326). It is depression sufferers who are most likely to attempt suicide (56–87%). (E. Isometsa [32]; Y. Kawashima et al. [33]). In Poland, around 20% of people with depression die following suicide (Kielan et al. [34]).

Results of studies on timing of suicidal behavior in particular populations (for example, patients hospitalized after suicidal poisoning and suicidal behavior recorded in national police databases) are based on numerical data that do not take into account the impact of other variables, e.g., demographic or mental health-related. Numerous studies confirm a strong relationship between suicidal behavior and the occurrence of depressive disorder, for instance, it has been found that 7–15% of patients with depressive disorders die by suicide (Wasserman and Wasserman [35]). The current study undertakes analyses of fluctuations in search interest for “suicide” and “depression” for particular days of the week and times of day using the Internet as a data source and takes into account depression as a suicide risk factor. Detailed objectives (research problems) include the comparison of trends in the increase in the number of hits regarding the terms “depression” and “suicide” depending on the time of day and the following day of the week, and the search for a relationship between the studied phenomena. Moreover, differences in the described trends between four countries (Poland, Germany, Great Britain, Italy) were analyzed.

## 2. Research Method

Google Trends is a tool that allows the user to measure the popularity of search terms with regard to geography and time. It uses data from the Google search engine. By using this service, we can primarily analyze the search trends for individual keywords or phrases in selected time periods. Data analysis refers to the number of searches for a given term entered on Google in relation to the total number of searched terms in the selected time period. Recently, more and more research is based on the use of this inference tool, in particular in medical and economic sciences (J. Seung-Pyo et al. [36]; M.H. Ginsberg et al. [37]. Based on the number of searches, we can determine the popularity of e.g., a disease or study the financial market (T. Preis [38]). The number of hits for selected terms in the Google search engine can also be used to analyze the interpretation of early warning. In Google Trends, the data in the chart are normalized and presented on a scale from 0 to 100. It does not represent the absolute search numbers for the given term. The study we conducted consisted in recording, on a weekly basis, the level number of search interest concerning the relevant keywords using Google Trends (GT). The study was carried out over 47 weeks starting in week 40th of 2021, every Monday at 8:00 a.m. taking into account the time zone. The gtrends package of the R program was used to read the data. The data on the particular queries were collected in a spreadsheet. Then the arithmetic mean of the percentage of hits was calculated for each hour. The test procedure is presented below.

1. The time range is the same for all surveyed territories.
2. We provide information on the researched terms for each territory respectively.
3. Graphs of averaged data indicate the presence of seasonality. We used the Python program to create the charts (version: 3.10) and the function seasonal-decompose from the statsmodels.tsa.seasonal library.
4. Each week we have 168 observations, which have been divided into seven subgroups corresponding to the consecutive days of the week. The first subgroup (observations indexed 0–23) corresponds to hits from Monday 8:00 a.m. to Tuesday 7:00 a.m., the second subgroup corresponds to hits from Tuesday 8:00 a.m. to Wednesday 7:00 a.m. and so on. In each of the subgroups we determine the maximum of hits assuming that if, in addition to the maximum value, there is a value not less than one in a given group, we indicate both values as the maximum.
5. The tables were exported to EXCEL and we used built-in functions to analyze the data. Additionally, columns indicating the appropriate days and hours of observation were imported. It needs to be noted that the entry “Mo 0:00” means midnight from Sunday to Monday.
6. Additionally, xlsx files with all data collected from a given period are attached. The csv files were combined into a data.frame object and then exported to an xlsx file using Python version 3.10.

## 3. Results

We will now compare hits for: “depression” and “suicide” for particular countries.

### 3.1. Poland

We start from the analysis of hits “depression” (PL: “depresja”) and “suicide” (PL: “samobójstwo”) for the territory of Poland. Summaries are provided in Table 1 and Table 2 below.

**Figure 1:**
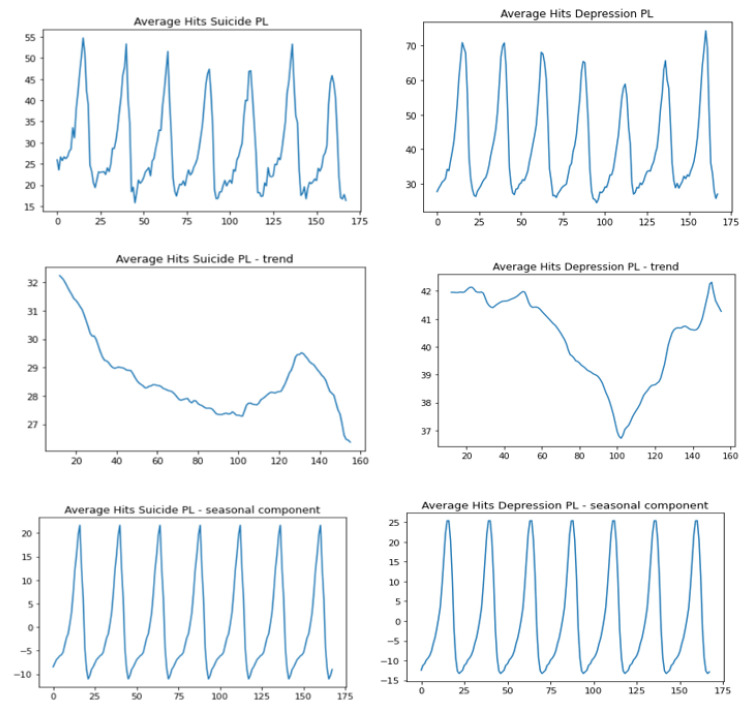
Hits “suicide” and “depression” for Poland–trend-seasonal decomposition.

The graphs made with the seasonal-decompose Python function from the statsmodels.tsa.seasonal library are shown in Figure 1. The first line shows the graph for the mean searches for “suicide” and “depression”, the second line of the graph for the trend component, and the third line for the seasonal component.

#### Conclusions

1. The search interest for “depression” and “suicide” increases at the turn of the day.
2. The average search interest for the term with regard to the day of the week: “depression” and “suicide”.
  - On Mondays and Tuesdays the peak search interest falls on the same time: between 22:00 and 23:00 (Monday) and between 23:00 and midnight from Tuesday to Wednesday.
  - On Wednesdays the search interest for “depression” is the highest between 21:00 and 23:00 while for “suicide” between 23:00 and 0:00 (midnight from Wednesday to Thursday).
  - We observe a similar relationship in the night from Thursday to Friday: search interest for “depression” is higher earlier (22:00–0:00), while for “suicide” at midnight, that is between 23:00 and 0:00.
  - In the night from Friday to Saturday the peak search interest for both terms falls on the time between 22:00 and 0:00.
  - A similar relationship is observed in the night from Saturday to Sunday: the peak search interest is between 23:00 and 0:00.
  - In the night from Sunday to Monday the term “depression” is mostly searched for between 23:00 and 0:00, which is later than “suicide” whose peak search interest is between 22:00 and 23:00.
3. Trend analysis
  - The trend for the term “suicide” is on a downward trend from the beginning of the week declining to a trough around Friday noon, then it increases until Saturday evening (around 18:00), then declines again.
  - For the term “depression” there is a downward trend until around Friday noon, then the trend increases until Sunday afternoon, then declines on Sunday evening.

### 3.2. Germany

We now turn to the analysis of hits “depression” (GER: “Depression”) and “suicide” (GER: “Selbstmord”) for the territory of Germany. Summaries are provided in Table 3 and Table 4 below.

**Figure 2:**
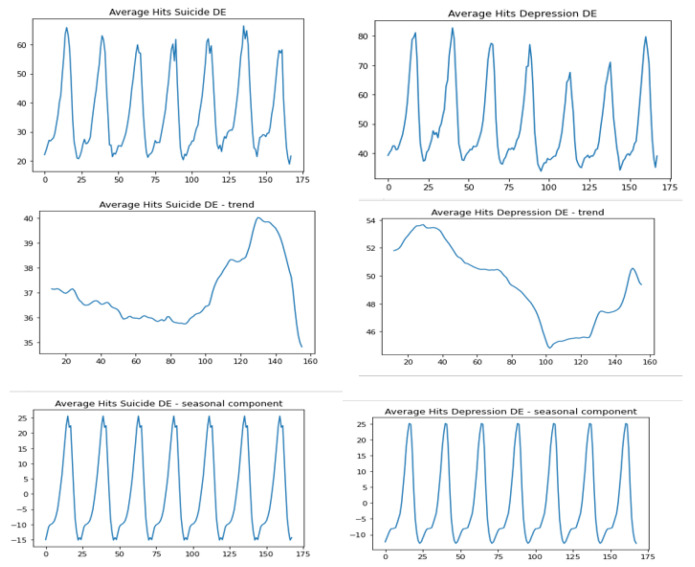
Hits “suicide” and “depression” for Germany–trend-seasonal decomposition.

The respective graphs for the averaged data from the territory “Germany”, the seasonal component and the trend component are presented in Figure 2.

#### Conclusions

1. The search interest for “depression” and “suicide” increases at the turn of the day
2. The average search interest for the term with regard to the day of the week: “depression” and “suicide”. The peak search interest for the term “depression” usually occurs later than that for the term “suicide”.
  - On Mondays the peak search interest for the term “suicide” falls between 22:00 and 23:00, while for the term “depression” it is two hours later: on Tuesday between midnight and 01:00.
  - On Wednesdays the search interest for the term “depression” is the most intense between 23:00 and midnight, while for the term “suicide” the peak search interest is observed earlier: on Tuesday between 22:00 and 23:00.
  - A similar relationship is observed in the night from Wednesday to Thursday: the search for the term “depression” intensifies between 23:00 and 1:00, while for the term “suicide” it is one hour earlier, that is between 22:00 and 23:00.
  - In the night from Thursday to Friday the peak search interest is observed in the reverse order: most search for “depression” is recorded on Thursday between 23:00 and 0:00, while the highest interest for the term “suicide” is noted on Friday between 0:00 and 1:00.
  - In the night from Friday to Saturday again the peak search interest is observed for the term “suicide” first (Friday: 22:00-23:00), and for the term “depression” two hours later, on Saturday between midnight and 1:00.
  - The same order is observed in the night from Saturday to Sunday: the increased intensity for the term “suicide” is seen on Saturday between 22:00 and 23:00, while for the term “depression” it is on Sunday between 1:00 and 2:00.
  - In the night from Sunday to Monday the term “depression” is most searched between 23:00 and 0:00, which is later than the term “suicide” whose peak search interest falls on the hour between 22:00 and 23:00.
3. Trend analysis
  - The trend for the term “suicide”: the downward trend occurs from Monday to Thursday evening hours (20:00–22:00), then increases until midnight from Saturday to Sunday, and then continues to decline again.
  - For the term “depression” there is also a downward trend from Thursday evening (between 19:00 and 21:00) until noon on Friday, which then increases until Sunday evening hours (19:00–21:00).

### 3.3. Italy

We now turn to the analysis of hits “depression” (IT: “depressione”) and “suicide” (IT: “suicidio”) for the territory of Italy. Summaries are provided in Table 5 and Table 6 below.

**Figure 3:**
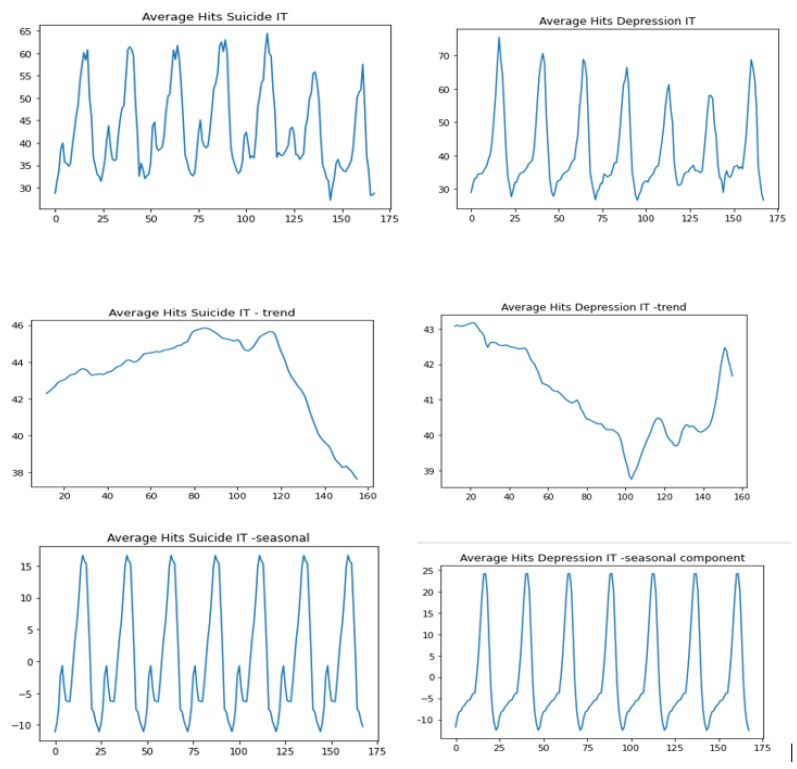
Hits “suicide” and “depression” for Italy–trend-seasonal decomposition.

The respective graphs for the averaged data from the territory “Italy”, the seasonal component and the trend component are presented in Figure 3.

#### Conclusions

1. The search interest for “depression” and “suicide” increases at the turn of the day.
2. The average search interest for the term with regard to the day of the week: “depression” and “suicide”. The peak search interest for the term “depression” usually occurs later than that for the term “suicide”. The exception is the night from Saturday to Sunday.
  - On Mondays the peak search interest for the term “suicide” falls on the hours between 22:00 and 1:00 Tuesday, while for the term “depression” it is two hours later: between midnight and 01:00 Tuesday.
  - In the night from Tuesday to Wednesday the search interest for the term “depression” is the highest between 00:00 and 01:00, while for the term “suicide” the peak interest is seen earlier: on Tuesday between 21:00 and 0:00.
  - A similar relationship is observed in the night from Wednesday to Thursday: the highest search interest for the term “depression” is between 23:00 and 1:00, while for the term “suicide” it is two hours earlier and falls on Wednesday between 21:00 and 23:00.
  - In the night from Thursday to Friday the peak search interest for the term “depression” falls on Friday between 0:00 and 1:00, while for the term “suicide” the most intense interest is observed between 22:00 on Thursday and 1:00 on Friday.
  - In the night from Friday to Saturday the peak search interest is observed for the term “suicide” first again (Friday 22:00–23:00), and for the term “depression” it is two hours later, on Saturday from midnight to 1:00.
  - The same order is observed in the night from Saturday to Sunday: the increased intensity for the term “suicide” is seen on Saturday between 22:00 to 0:00, while for the term “depression” it is between 23:00 and 1:00.
  - In the night from Sunday to Monday the term “depression” is most searched between 23:00 and 0:00, which is earlier than the term “suicide”, whose peak search interest is observed between 00:00 and 01:00 on Monday.
3. Trend analysis
  - The trend for the term “suicide” is slightly increasing until Saturday morning (between 8:00 and 10:00), then we observe a fairly sharp decline.
  - For the term “depression” there is a downward trend until noon on Friday, then we observe an increase in interest until Sunday evening.

### 3.4. Great Britain

We now turn to the analysis of hits “depression” and “suicide” for the territory of Great Britain. Summaries are provided in Table 7 and Table 8 below. The time indicated in brackets is the two-hour difference between the observed average time and UTC.

**Figure 4:**
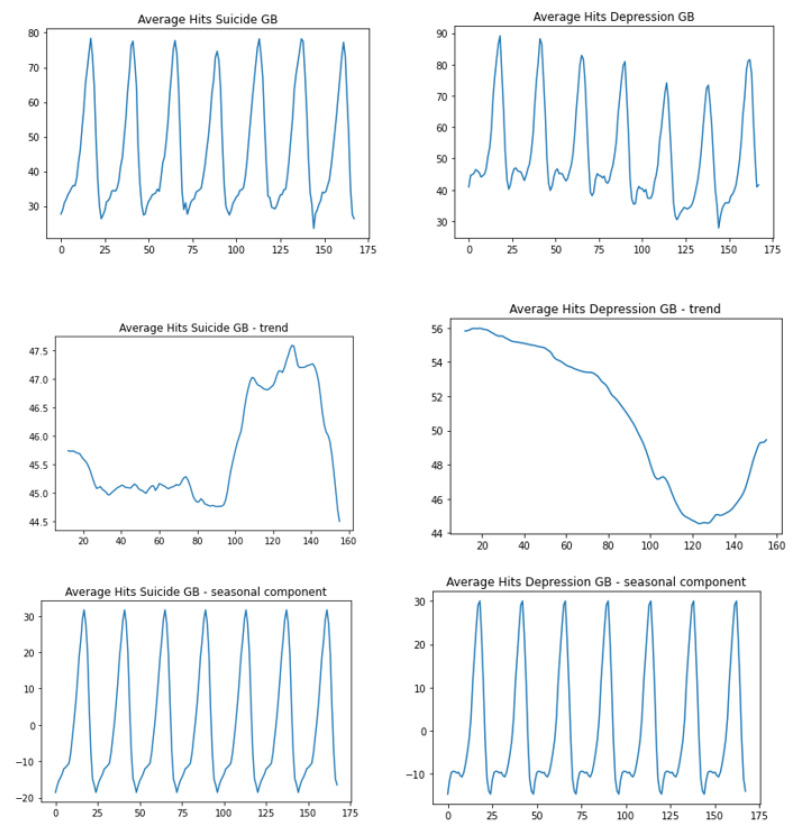
Hits “suicide” and “depression” for Great Britain–trend-seasonal decomposition.

The respective graphs for the averaged data from the territory “Great Britain”, the seasonal component and the trend component are presented in Figure 4.

#### Conclusions

1. The average search interest for the term with regard to the day of the week: “depression” and “suicide”. The peak search interest for the term “depression” usually occurs later than the peak search interest for the term “suicide”.
  - Time of the highest search intensity for the term “suicide” is the same everyday and falls on the hour between 22:00 and 23:00.
  - Daily peak search interest for the term “depression” is observed at the same time as the peak reached for “suicide” (on Wednesdays, Thursdays and the night from Saturday to Sunday), or later, between 23:00 and 0:00 on Mondays, Thursdays, Fridays and Saturdays.
2. Trend analysis
  - For the term “suicide” we observe a downward trend until Saturday morning (8:00–10:00), then the trend increases.
  - For the term “depression” we observe a downward trend until Friday morning, then increased interest until Sunday morning (5:00–7:00), and a downward trend again at the end of the week.

## 4. Discussion and Conclusions

In the four analysed countries, the peak search interest for the terms “suicide” and “depression” was observed in the late evening and early night hours, i.e., between 22.00 and 1.00. These queries were most frequent at the end of the week (at weekends), and this trend starts to decline from Monday onwards. The exception was Italy, where the two search terms reached their peaks on Fridays, with the search frequency gradually decreasing at weekends and increasing again from late Sunday evening onwards.

Further, our study found that in Poland, the peak search interest for the term “depression” occurred earlier than that for the term “suicide” on each day of the week, with the exception of the night from Sunday to Monday. In Germany, the highest search intensity for the term “suicide” occurs earlier that that for the term “depression” on all days of the week except for the night from Thursday to Friday. A similar regularity was observed in Italy: the highest search intensity for the term “suicide” occurred earlier that that for the term “depression” on all days of the week except for the Sunday night. In Great Britain, the search interest for the term “suicide” reached its peak earlier that that for the term “depression” on any day of the week.

Results of our Google Trends study on online queries on “depression” and “suicide” show a similarity with results of studies on the frequency of suicide and suicide attempts on particular days of the week and hours of the day. At the same time, searching terms “suicide” and/or “depression” may not be equivalent to the intention to engage in suicidal behavior. On the contrary, a person may be using the Internet to look for help or is interested in the phenomenon of suicide. However, the population of people who enter these search queries, also includes those who contemplate a suicide attempt or suicide. From this perspective, the practical value of the Google Trends research conducted in this study cannot be underestimated. Data regarding days of the week and times of day and night with intensified search interest for "suicide" and "depression", can inform operation of helplines. Our study shows that in analyzed countries, suicide prevention helplines should be particularly available around the weekend (Saturday, Sunday, Monday), and in the late evening and early night (between 22.00 and 1.00).

Our study has a number of limitations. The research is more observational than explanatory, and any proposed explanations for the observed similarities and differences between countries included in the analysis can only be speculative. We did not explore individual factors, such as levels of current suicidal ideation, previous suicidal behaviour, psychosocial and cultural factors (e.g., social classes or imitation Maiese [39]), support available, and help seeking. Further, due to the limited time period (47 weeks), our Google Trends research study does not provide information on the association between online searches for terms “suicide” and “depression” and the seasonality of suicide attempts and suicides in Poland, Germany, Italy and Great Britain. These limitations can be addressed by future explanatory research focusing on individual differences, e.g., involving individuals conducting online searches and asking them about motivation around particular search terms, location, and timing of searches.

## Figures and Tables

**Table 1:** Average hits–depression (PL).

Mo	Wed	Wed	Thu/Fri	Fri/Sat	Sun	Sun
23:00	00:00	22:00–23:00	23:00–00:00	23:00–00:00	00:00	00:00

**Table 2:** Average hits–suicide (PL).

Mo	Wed	Thu	Fri	Fri/Sat	Sun	Sun
23:00	00:00	00:00	00:00	23:00–00:00	00:00	23:00

**Table 3:** Average hits–depression (DE).

Tue	Wed	Thu	Fri	Sat	Sun	Mon
01:00	00:00	00:00–01:00	00:00	01:00	02:00	00:00

**Table 4:** Average hits–suicide (DE).

Mo	Tue	Wed	Fri	Fri	Sat	Sun
23:00	23:00	23:00	01:00	23:00	23:00	23:00

**Table 5:** Average hits–depression (IT).

Tue	Wed	Thu	Fri	Sat	Sun	Mon
00:00	01:00	00:00–01:00	01:00	01:00	00:00–01:00	00:00

**Table 6:** Average hits–suicide (IT).

Mon/Tue	Tue/Wed	Wed/Thu	Thu/Fri	Fri	Sat/Sun	Mon
23:00–01:00	22:00–0:00	22:00–0:00	23:00–1:00	23:00	23:00–0:00	01:00

**Table 7:** Average hits–depression (GB).

Tue	Wed	Thu	Fri	Sat	Sun	Sun/Mon
02:00	01:00	01:00	02:00	02:00	02:00	01:00–02:00
(00:00)	(23:00)	(23:00)	(00:00)	(00:00)	(00:00)	(23:00–1:00)

**Table 8:** Average hits–suicide (GB).

Tue	Wed	Thu	Fri	Sat	Sun	Mon
01:00	01:00	01:00	01:00	01:00	01:00	01:00
(23:00)	(23:00)	(23:00)	(23:00)	(23:00)	(23:00)	(23:00)

## Data Availability

In the article the daily data of Google Trends https://trends.google.pl/trends/?geo=PL (Access dates: every seven days between 11 October 2021 and 29 August 2022) were used. The study was carried out over 47 weeks starting in week 40th of 2021, every Monday at 8:00 a.m. taking into account the time zone. The data were downloaded 47 times, every seven days starting from 11 October 2021 and ending at 29 August 2022.

## Sample Availability

Samples of the compounds are available from the authors.
